# Iron-sulfur chemistry can explain the ultraviolet absorber in the clouds of Venus

**DOI:** 10.1126/sciadv.adg8826

**Published:** 2024-01-03

**Authors:** Clancy Zhijian Jiang, Paul B. Rimmer, Gabriella G. Lozano, Nicholas J. Tosca, Corinna L. Kufner, Dimitar D. Sasselov, Samantha J. Thompson

**Affiliations:** ^1^ Department of Earth Sciences, University of Cambridge, Downing St., Cambridge CB2 3EQ, UK.; ^2^ Cavendish Laboratory, University of Cambridge, JJ Thomson Ave, Cambridge CB3 0HE, UK.; ^3^ Harvard-Smithsonian Center for Astrophysics, Harvard University, 60 Garden Street, Cambridge, MA 02138, USA.

## Abstract

The clouds of Venus are believed to be composed of sulfuric acid (H_2_SO_4_) and minor constituents including iron-bearing compounds, and their respective concentrations vary with height in the thick Venusian atmosphere. This study experimentally investigates possible iron-bearing mineral phases that are stable under the unique conditions within Venusian clouds. Our results demonstrate that ferric iron can react with sulfuric acid to form two mineral phases: rhomboclase [(H_5_O_2_)Fe(SO_4_)_2_·3H_2_O] and acid ferric sulfate [(H_3_O)Fe(SO_4_)_2_]. A combination of these two mineral phases and dissolved Fe^3+^ in varying concentrations of sulfuric acid are shown to be good candidates for explaining the 200- to 300-nm and 300- to 500-nm features of the reported unknown UV absorber. We, therefore, hypothesize a rich and largely unexplored heterogeneous chemistry in the cloud droplets of Venus that has a large effect on the optical properties of the clouds and the behavior of trace gas species throughout Venus’s atmosphere.

## Teaser

Iron and sulfur can interact in the droplets of Venus’s clouds to form minerals, which absorb ultraviolet light.

## INTRODUCTION

The clouds of Venus hold several mysteries. They extend from 48 km to roughly 65 km (*1*) and provide a transition region between the lower atmosphere (<48 km) that is dominated by thermochemistry and dynamics (*2*) and the upper atmosphere (>65 km), for which photochemistry and dynamics are relevant (*3*). To understand the chemical cycles between the Venusian atmosphere and its volcanic surface, and to correctly interpret potential biosignatures (*4*), an increasing research effort has been dedicated to generating a complete model framework of Venusian atmosphere (*5*, *6*). Addressing the complex physicochemical conditions within the cloud layers underpins the interpretation of new observational data expected from upcoming missions that target the Venusian atmosphere (*7*).

Mostly the only available data for the composition of the clouds and the gas-phase chemistry in the region 48 to 60 km (except for H_2_SO_4_ vapor) were collected in situ by probes [see Appendix A of Rimmer *et al.* (*6*), which contains an extensive list of the observed gas-phase concentrations and references]. These data reveal a wealth of plausible physical and chemical processes that are poorly constrained. These include, but are not limited to, the depletion of sulfur dioxide and water in the clouds (*6*, *8*), an unknown ultraviolet (UV) absorber at the top of the clouds (*1*, *9*), discrete size distributions of aerosol particles (*10*), redox disequilibrium chemistry (*5*, *11*, *12*), and the possible presence of a cloud layer (47 to 52 km), tentatively indicated by VEGA-2 x-ray data, where phosphorus is at least as abundant as sulfur (*13*, *14*). Of import, current models of atmospheric chemistry on Venus are unable to explain a number of observations, such as the behavior of carbonyl sulfide (OCS) and sulfuric acid (H_2_SO_4_) vapor below the clouds, the behavior of water vapor and sulfur dioxide (SO_2_) in and above the clouds, and the lack of O_2_ above the clouds [see Bierson and Zhang (*5*) for a helpful summary of these four outstanding problems]. Some of these features are better supported than others, and in particular, the tentative observations of redox disequilibrium chemistry and abundant phosphorus in the clouds require observational confirmation.

This investigation limits its scope to the unknown absorbers in the Venusian clouds that have contributed to UV and blue light absorbance observed at wavelengths extending from 200 to 300 nm (*15*, *16*) and 300 to 500 nm (*16*–*18*). An increasing volume of literature supports several candidate absorbers that are products of the iron and/or sulfur cycles in the Venusian atmosphere; these include ferric chloride (FeCl_3_) (*19*), amorphous sulfur (*20*), disulfur monoxide (S_2_O) (*20*, *21*), ammonium pyrosulfate [(NH_4_)_2_S_2_O_5_] (*22*), and sulfur monoxide dimer (OSSO) (*23*). The nature, abundance, and distribution of these candidate absorbers, however, remain a matter of debate (*17*, *24*, *25*). The readers are referred to the Supplementary Materials for further discussion regarding the suitability of these candidates.

Both iron and sulfur have been reported in the Venusian clouds by various probes [x-ray fluorescence; (*13*, *26*)] and may have been detected in the atmosphere below the clouds by the Pioneer missions (*27*). Gas chromatography analysis on board VEGA 1 and 2 probes has, in addition, inferred elemental sulfur in the clouds (*28*). These observations have motivated studies dedicated to exploring how iron and sulfur may interact in a Venus analog environment (*29*, *30*), and their products have been proposed as candidate absorbers in the clouds of Venus (*31*). The exact nature and speciation of these elements and compounds, however, remain unclear. This is because iron-sulfur chemistry is subject to numerous poorly constrained parameters in the Venusian clouds (e.g., water activity, pH, oxidation states, pressure, and temperature). In terrestrial environments, the structural and thermodynamic data of iron-sulfur–bearing minerals have been extensively documented due to their importance in modeling and remedying efforts regarding acid mine drainage (*32*–*35*). The detection of multiple ferric sulfate phases on the Martian surface during orbital remote sensing and rover missions has also sparked laboratory spectroscopic investigations (*36*–*38*). Whether these minerals are stable under the harsh but variable Venusian atmospheric conditions, thus potentially contributing to the observed UV absorbance, remains unanswered.

Here, we explore the reaction of Fe^3+^ with sulfuric acid that produces various ferric sulfate minerals including rhomboclase [(H_5_O_2_)Fe(SO_4_)_2_·3H_2_O] and acid ferric sulfate [AFS; (H_3_O)Fe(SO_4_)_2_]. The stability and distribution of rhomboclase and AFS are dictated by water activity (*39*). In Venusian clouds, water activity can be correlated to the concentrations of sulfuric acid and the atmospheric height. This study presents the UV measurement of synthetic rhomboclase and AFS to demonstrate that a combination of these two minerals, in addition to dissolved iron, can adequately explain the observed UV absorbance. These results and implications reveal and highlight the heterogeneity in the physicochemical and optical properties of Venusian clouds, which must be incorporated in full atmosphere models that will aid in future exploration missions.

## RESULTS

Depending on the wt % H_2_SO_4_, pressure, and temperature, Fe^3+^ will react with sulfuric acid to form anhydrous ferric sulfate [Fe_2_(SO_3_)_3_], AFS [(H_3_O)Fe(SO_4_)_2_], and/or rhomboclase [(H_5_O_2_)Fe(SO_4_)_2_·3H_2_O)]. There may be pockets of Venus’s clouds with <50 wt % H_2_SO_4_ in the droplets and local water vapor concentration exceeding 1000 ppm (*40*). There, the stable iron-sulfur minerals could be copiapite [Fe^2+^Fe^3+^_4_(SO_4_)_6_(OH)_2_·20H_2_O] and other hydrated iron-sulfur minerals. These hydrated minerals, once formed, could have persisted in regions of elevated wt % H_2_SO_4_ for an observable period of time, thus contributing to the reported UV absorbance.

**Fig. 1.**
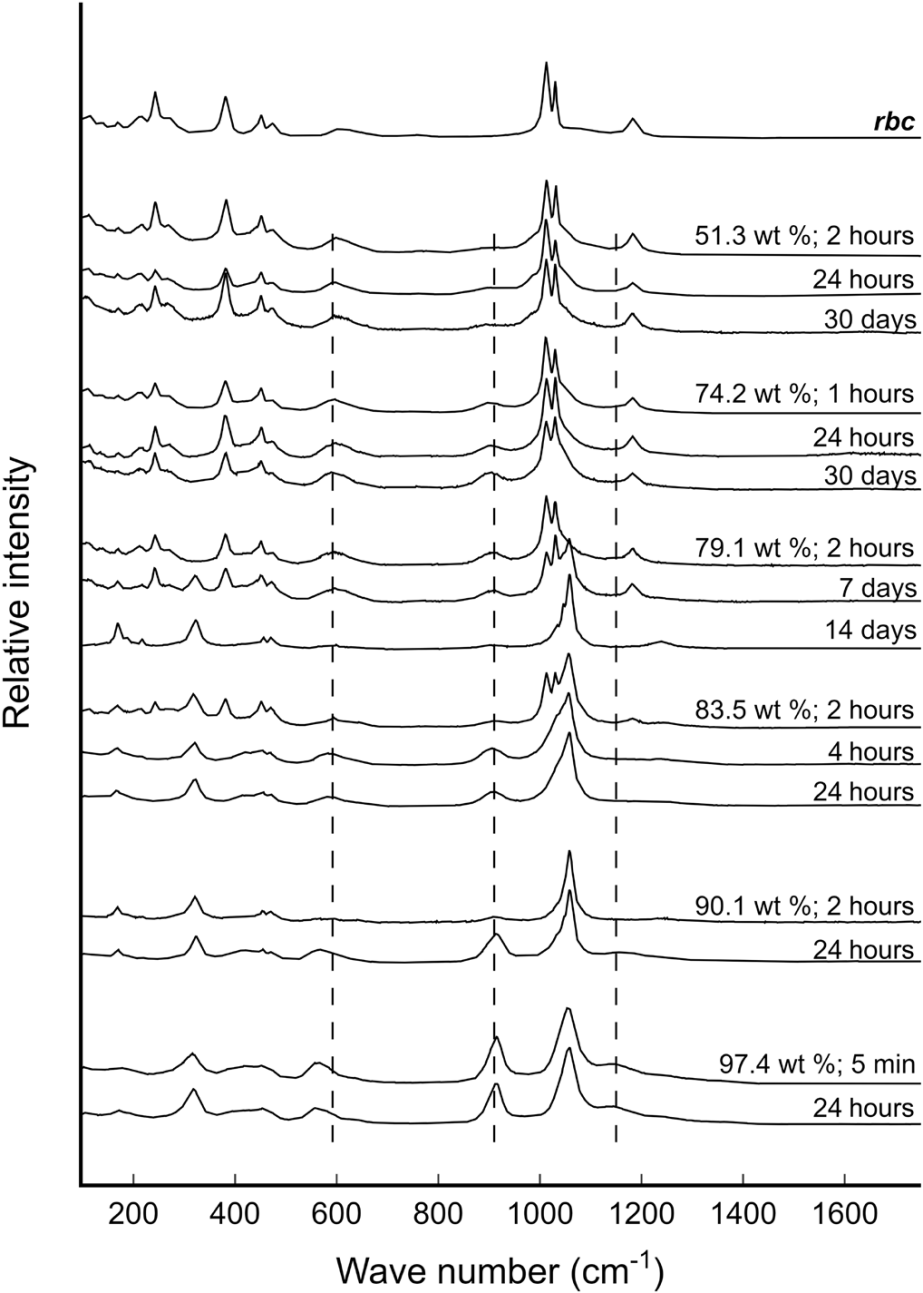
Raman shifts (532 nm) of rhomboclase (rbc) in varying wt % of sulfuric acid over time. rbc is stable in 74.2 wt % sulfuric acid over 30 days. However, rbc → acid ferric sulfate (AFS) transformation is completed within 14 days in 79.2 wt %, within 2 hours in 83.5 wt %, and within 5 min in 97.4 wt % sulfuric acid. Dilution of sulfuric acids due to rbc → AFS transformations is calculated based on the added solid mass. Dotted lines indicate Raman shifts contributed by sulfuric acid (fig. S3), and the relative intensities of sulfuric acid peaks are attributed to the solution-solid ratio and particle size at the focal point of the laser. For details, including further Raman and XRD to establish the mineralogy, see Materials and Methods and the Supplementary Materials.

To test the relative stability of Fe(III)-sulfate minerals in the Venusian clouds, synthesized copiapite and rhomboclase (see Materials and Methods) were suspended in sulfuric acid concentrations from 50 to 98 wt %, and their mineralogy was monitored over a period of 30 days at room temperature and atmospheric pressure. This wide range of wt % H_2_SO_4_ can be correlated to Venusian atmospheric height, encompassing the large model uncertainties as well as the possibility of localized, low wt % H_2_SO_4_ pockets in the clouds (see Materials and Methods). Time-series in situ Raman analyses of suspended solids identified two transitions. The first is the transformation of copiapite to rhomboclase in 51.3 wt % H_2_SO_4_ within 9 days (fig. S6). The second is the transformation of rhomboclase to AFS in 79.1 wt % H_2_SO_4_ within 14 days (Fig. 1). These two transformations are in broad agreement with thermodynamic predictions and previous laboratory observations (*32*, *35*, *41*, *42*), and the rates of transformations are expected to escalate with increasing wt % H_2_SO_4_. Notably, near the edge of rhomboclase stability in 74.2 wt % H_2_SO_4_, the rhomboclase → AFS transformation was so slow that rhomboclase remained the only mineral phase after 30 days (Fig. 1).

**Fig. 2.**
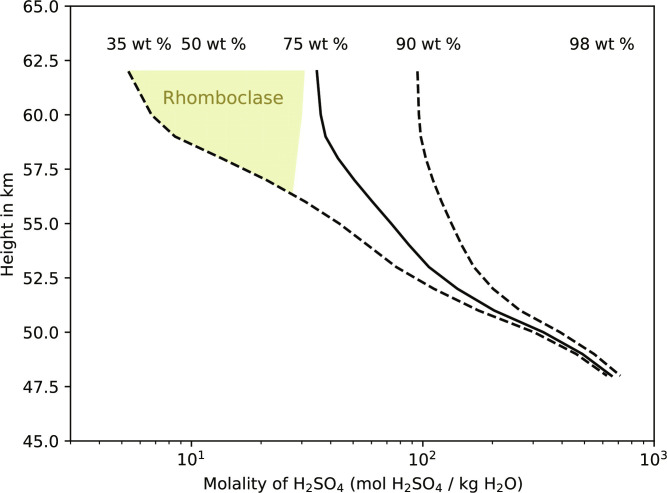
H_2_SO_4_ molality of Venus’s clouds. The molality of H_2_SO_4_ in the cloud droplets of Venus (mol H_2_SO_4_/kg H_2_O) as a function of atmospheric height (km) is based on the nominal model predictions of Dai *et al.* (*51*) (solid line), with errors based on observational uncertainties (*43*) (dashed lines). Water activity estimation as a function of H_2_SO_4_ concentration and temperature was achieved using E-AIM model detailed by Wexler and Clegg (*59*). The stability field of rhomboclase-AFS was determined by assuming a temperature-independent standard state heat capacity of this reaction due to limited thermodynamic data (see Materials and Methods). Equivalent eight percentages are given. The yellow-shaded region is where rhomboclase is stable. Outside this region, within the bounds delineated by the dashed lines, rhomboclase is not thermodynamically stable and should eventually be converted into AFS.

The modeling and experimental results presented here indicate that the dominant iron-sulfur species in the Venusian clouds (∼70 to 98 wt %; 62 to 48 km atmospheric height) could be a combination of AFS and rhomboclase (Figs. 1 and 2). This combined mineral stability range is consistent with the low (∼30 ppm) to mid-range (∼300 ppm) in situ H_2_O concentrations observed in the clouds (*43*). Although the possibility of more hydrate iron-sulfur minerals is not ruled out, the short-lived presence of copiapite in H_2_SO_4_ ≥50 wt % (Fig. 2) suggests that its distribution in Venusian clouds is extremely limited or negligible.

**Fig. 3.**
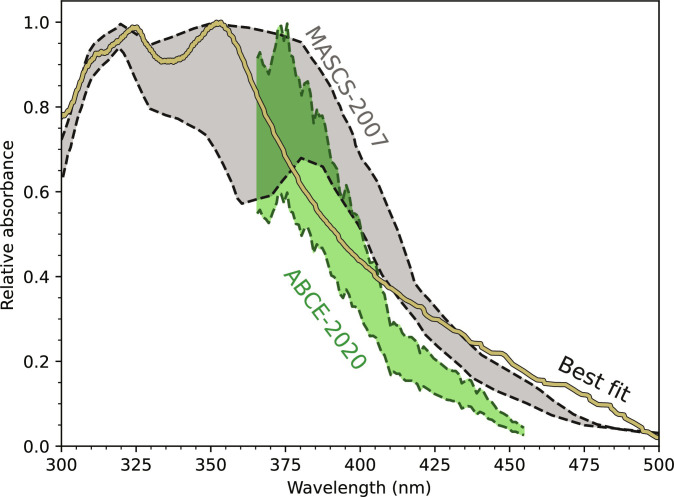
Iron sulfur candidate for the 365-nm UV absorber. Relative ultraviolet-visible absorbance as a function of wavelength (nm) for the 365-nm component of the UV absorber. MESSENGER/MASCS Observations of the UV absorber with variation and uncertainty (*17*) (black dashed lines and gray region) and a combination of Akatsuki, Bepi-Columbo, and Earth-based measurements [ABCE-2020, (*18*)] are compared to our best-fit results. The best fit is a linear combination of roughly equal parts 1 wt % rhomboclase in 74 wt % H_2_SO_4_ and 1.25 wt % AFS in 84 wt % H_2_SO_4_, suggesting contributions from droplets with different sulfuric acid concentrations. For details, see Materials and Methods and the Supplementary Materials.

**Fig. 4.**
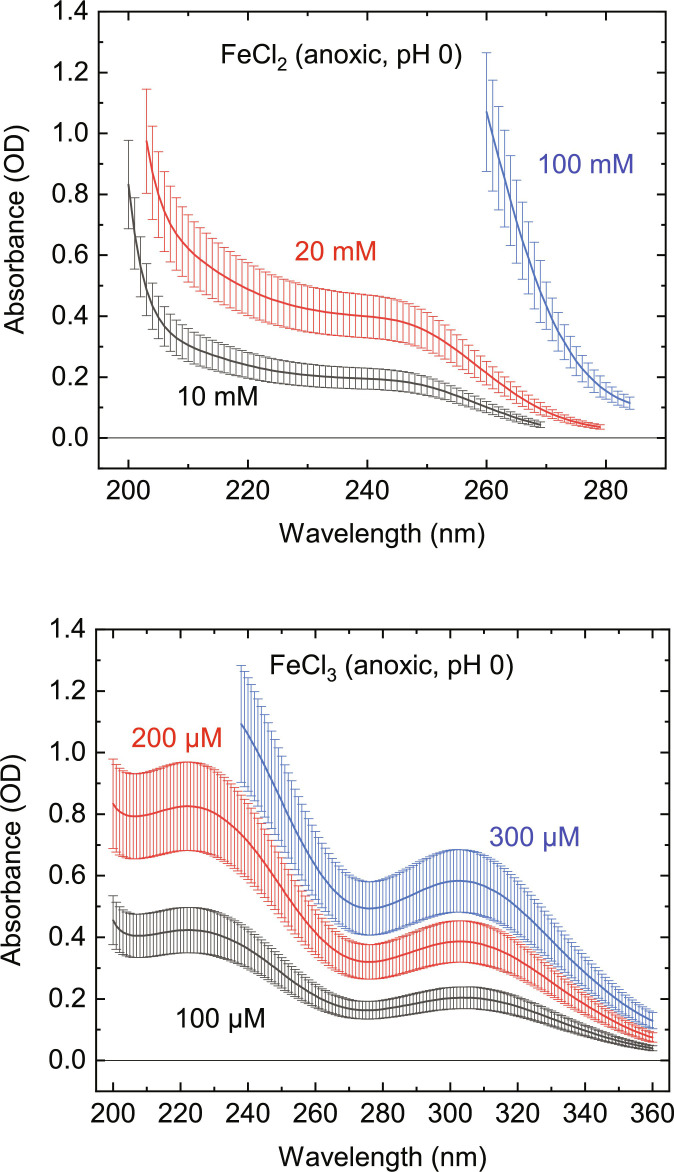
UV/Vis absorbance spectra of anoxic Fe^2+^ and Fe^3+^. Absorbance [decadal optical depth (OD)] as a function of wavelength (nm) for FeCl_2_ (top) and FeCl_3_ (bottom) at different concentrations (pH 0).

Aliquots of various wt % H_2_SO_4_ loaded with optically optimum amounts of suspended rhomboclase and AFS were analyzed for their UV absorbance (see Materials and Methods), which yielded reasonable agreement with the observed UV absorber in the clouds of Venus (Fig. 3). In addition, the UV absorbance of Fe^3+^ solution (using FeCl_3_ as starting material) produces a good match with the 200- to 300-nm absorber (see Fig. 4). This lends support to the hypothesis advocated by Kuiper (*44*), Zasova *et al.* (*45*), and Krasnopolsky *et al.* (*19*).

## DISCUSSION

Despite the thermodynamic prediction of rhomboclase stability being limited to H_2_SO_4_ below ∼75 wt % (Fig. 2) (*35*), time-series Raman analysis highlighted the notably slow rhomboclase → AFS reaction kinetics in acid concentrations below ∼80 wt % (Fig. 1). Considering the time frame of convection and aerosol settling in the mid to upper Venusian clouds to be likely less than 30 days (*46*), rhomboclase, once formed, could have persisted in regions of higher H_2_SO_4_ concentration and contributed to the observed UV absorbance. In other words, the distribution of rhomboclase in the Venusian clouds may extend beyond the thermodynamic prediction due to the slow rhomboclase → AFS reaction kinetics in H_2_SO_4_ ∼80 wt % (i.e., down to 55 km atmospheric height as shown in Fig. 2). It is worth pointing out that rhomboclase will only form in the clouds of Venus in the first place if some regions of the cloud droplets have disequilibrium wt % H_2_O, or wt % H_2_O in equilibrium with ≳100 ppm concentrations of water vapor. These concentrations are higher than the ground-based below-cloud observations of 30 ppm, but are consistent with the range of measured values discussed by Ignatiev *et al.* (*43*).

The presence of AFS and rhomboclase is consistent with observational and model constraints on the concentration of H_2_SO_4_ in the clouds of Venus, when accounting for uncertainties. Models, such as those of Krasnopolsky (*47*), show models of H_2_SO_4_ concentrations between ∼70 and 90 wt %, with the lower values at the cloud tops. Krasnopolsky favors a minimum H_2_SO_4_ concentration at the cloud tops of just below 75 wt % at 60° latitude. Observations are also consistent with a range of constraints between 70 wt % and 96 wt %, varying with atmospheric height, with lower sulfuric acid concentrations favored higher in the clouds (>60 km) (*48*, *49*). Most of these constraints are based on comparisons of observed reflectivity with refractive index data for H_2_SO_4_-H_2_O systems at varying concentrations. It is worth noting that not all observationally constrained indices of refraction are compatible with pure H_2_SO_4_-H_2_O droplets (*50*). The range of H_2_SO_4_ concentrations we adopt for this study (as presented in Fig. 2) is from Dai *et al.* (*51*), where observational uncertainties are accounted for (see Materials and Methods).

The specific shape of the UV absorbance is influenced by the concentrations of both the mineral phase and sulfuric acid. This may be attributed to other minority mineral phases participating at different sulfuric acid concentrations that are not characterized in this investigation, or because the concentrations themselves affect the shape of the UV absorbance. It has been reported that UV absorbance varies with solution ionic strength (*52*). Further experimental studies on characterizing minority mineral phases that may exist in cloud droplets will greatly improve our quantitative constraints on Venusian atmospheric chemistry.

The iron that is required to explain the UV absorber amounts to 1 to 2 wt %, which is similar to the iron content estimated by Krasnopolsky (*19*) and required for FeCl_3_ by Zasova *et al.* (*45*). Krasnopolsky (*19*) assumes the flux is in the form of Fe_2_Cl_6_ vapor, although there are thermodynamic arguments that the dominant gas-phase iron species is FeCl_2_ or Fe_2_Cl_4_, and that their gas-phase abundances are negligible (*53*). The iron (III) chloride that enters into the cloud droplets will be rapidly converted to AFS in 90 wt % H_2_SO_4_ (see the Supplementary Materials).

Iron could also be delivered by volcanism, drifting of dust on the surface or from cosmic dust (*54*). Very generous estimates of iron delivery (*4*) amount to iron fluxes of ≲10^−15^ g cm^−2^ s^−1^, corresponding to at most 10^−3^ wt % iron in the clouds, a negligible amount compared to the flux estimates for iron-containing gas-phase species. It is difficult to constrain volcanic or other surface sources, because no observations currently provide any robust constraints to the presence or absence of iron in these sources.

The presence of Fe^2+^ and Fe^3+^ in the cloud droplets will sequester some SO_2_ into iron minerals, and this will contribute to the depletion of SO_2_ in the clouds. The amount of this depletion required to explain that the UV absorber, on the order of 1 to 2 wt %, is a tiny fraction of the total SO_2_ depletion (*6*). The sequestered sulfate will decompose to restore SO_2_ to the gas phase below the clouds (*27*, *53*), depending on the kinetics of iron sulfate decomposition.

The suspension of two minerals, acid iron sulfate and rhomboclase, along with dissolved Fe^3+^ in the liquid phase, can explain the observed UV absorption. Open questions about the source and the fate of the iron can be investigated by future missions, such as the DAVINCI mission (*7*). The Venus Mass Spectrometer (VMS) in particular, with its broad mass range of 2 to 550 Da, will potentially be able to detect iron-bearing species in the gas phase beneath the clouds, whether FeCl_2_, FeCl_3_, or their dimers. Proposed missions to interrogate aerosol compositions (*55*) could, in principle, identify the mineral phases we propose. Of equal importance, DAVINCI will be following the concentration of water vapor throughout the clouds and may better establish whether there are regions where droplets in equilibrium with the gas can form rhomboclase. If the iron sulfates rain out of the clouds as ferric sulfates, their degradation products could explain the anomalously high quantities of Fe^3+^-bearing minerals (*19*, *53*), inferred by the surface color observed by Venera (*56*). A future Venus lander mission would be required to test this hypothesis.

## MATERIALS AND METHODS

### UV spectrum of dissolved iron

Iron (II) chloride salt, iron (III) chloride salt, and sulfuric acid liquid (95 to 98 wt %) were purchased from Sigma-Aldrich (USA) at the highest available purity grade and used without further purification. Liquid chromatography–mass spectrometry (LC-MS) grade fresh water (LiChrosolv, Millipore Sigma, USA) was degassed with pure nitrogen gas for 30 min and then used to dissolve and dilute all samples anoxically in a glove box with O_2_ levels ranging from 30 to 200 parts per million (ppm).

MQuant pH-Indicator Strips were used to monitor the pH of the solutions as they were adjusted. Small amounts of sulfuric acid were added to both solutions until a pH of 0 was achieved. The pH of each solution was first adjusted at the highest concentration and then monitored with each dilution step. More sulfuric acid was added as needed to maintain a pH of 0.

The samples were placed in sealable spectrosil quartz cuvettes (9B-Q-10, Starna Cell’s, USA) with a sample depth of 10 mm. The volume of the sample in the cuvette was 0.8 ml. Samples were transferred to cuvettes anoxically. The cuvettes were cleaned with LC-MS–grade water between each dilution step. Absorbance spectra were recorded in triplicate at 23°C with a spectrophotometer (UV-1900, Shimadzu). Each measurement was done relative to blank water in the same type of cuvette. See Table 1 for a summary of the measurements.

**Table 1.** List of samples and measurement sequences for iron (II, III) chloride. Different concentrations of chloride are added to 0.8 ml of pure water, with H_2_SO_4_ added until the pH reaches zero.

Sample	Measurement sequence
Iron (II) chloride	100 mM (+ 20 μl H_2_SO_4_), 20 mM, 10 mM (+ 5 μl H_2_SO_4_)
Iron (III) chloride	10 mM (+ 2 μl H_2_SO_4_), 0.3 mM, 0.2 mM, 0.1 mM (+ 1 μl H_2_SO_4_)

### Mineral synthesis and characterization

To fully characterize the mineral stability of Fe-SO_4_^2^–H_2_O system over a broad range of sulfuric acid concentrations, we prepared copiapite [Fe^2+^Fe^3+^_4_(SO_4_)_6_(OH)_2_·20H_2_O] and rhomboclase [(H_5_O_2_)Fe(SO_4_)_2_·3H_2_O] following procedures detailed by Majzlan and Michallik (*33*) and Majzlan (*34*). Both minerals were prepared with Fe(III) sulfate hydrate powder [Fe_2_(SO_4_)_3_·*x*H_2_O; Fisher Chemical], sulfuric acid (98 wt %; AnalaR Chemicals), and deionized (DI) water. Because of the hygroscopic nature of Fe(III) sulfate hydrate powder, the exact hydration state was not determined but assumed to be the most common pentahydrate.

For copiapite syntheses, 15 g of Fe(III) sulfate pentahydrate was added to a borosilicate glass bottle (100 ml), followed by the addition of 15 ml of DI water. The Fe(III) sulfate powder instantly dissolved in water to form a dark brown solution and produced a yellowish-green slurry within 3 days. For rhomboclase syntheses, Fe(III) sulfate pentahydrate (15 g) was first dissolved in 24.45 ml of DI water followed by 6.8 ml of 98 wt % sulfuric acid. Upon acid addition, white precipitates were formed and resided at the bottom of the glass bottle within the acid portion. This white precipitate was likely anhydrous/AFS as a result of dehydration by 98 wt % sulfuric acid, and was completely dissolved after thoroughly mixing the solutions by shaking. The solutions were left in an incubator to slowly evaporate at 30°C for 2 weeks or until enough solids can be collected. Different from the slurry precipitates in copiapite syntheses, rhomboclase syntheses produced white crystalline hard encrustations on the glass bottle. Both precipitates were drained over fiberglass membranes (pore size ∼ 0.3 μm; Sartorius) without rinsing. It is observed that the use of both acetone and ethanol absolute (≥99.7%; SLS select) might cause mineral transformation into more hydrated phases.

Drained solids were mounted on custom-made polytetrafluoroethylene (PTFE) sample holders for powdered x-ray diffraction (XRD) analyses using a Bruker Diffracplus D8 Advance with a Mo Kα source (λ = 0.70930 Å) operated at 50.0 kV and 40.0 mA, scanned between 2.5 and 30 deg 2**Θ** at a step size of 0.02 deg (fig. S5). Raman shifts were obtained using HORIBA LabRam300 with 532-nm laser at ×50 magnification at 10 mW for solids (fig. S5) and 20 mW for solids suspended in sulfuric acid.

After confirming the mineralogy, both copiapite and rhomboclase were suspended in 10 ml of H_2_SO_4_ with concentrations varying from 50 to 98 wt %. An aliquot of 4 ml was pipetted in a 10-mm path-length quartz cuvette that can be mounted on a high-precision remote-controlled sample stage that allowed time-series Raman microscopy analysis directly on the suspended solids (Fig. 1). Raman shifts of the cuvette and blank sulfuric acids were also obtained to distinguish their peaks in the suspended solid analysis (fig. S7). The suspended solids were analyzed periodically over 30 days to monitor mineral stability.

Several extraction methods were carried out to obtain solids for powdered XRD analyses while retaining their mineralogy that is stable at high concentrations of H_2_SO_4_. Both rinsing with acetone and ethanol absolute resulted in the transformation of unstable AFS into rhomboclase. One plausible explanation is the protonation and dehydration of acetone (C_3_H_6_O) in high wt % H_2_SO_4_ that produce C_9_H_12_ and H_2_O (*57*), which subsequently hydrates AFS. Additionally, AFS might have reacted with humidity in the high airflow pulled by a vacuum pump. Because the residual H_2_SO_4_ exerts a negligible effect on XRD and Raman analyses of the solid materials, it is suggested that solid materials should only be drained on acid-resistant membranes (e.g., fiberglass) without vacuuming and analyzed on PTFE sample holders immediately. Raman analysis confirmed that there has not been mineralogical change before and after extraction + XRD analysis (a 10-min scan).

Because of the varying states of hydration and the degree of freedom of the protonated species in bonding configuration within the solid materials, x-ray diffractograms of copiapite, rhomboclase, and AFS can vary substantially in their relative peak intensities and position. This factor necessitates Rietveld refinement to fully characterize Miller indices and d-spacing (fig. S8). The synthetic materials fit well with the reported crystal structures (*32*, *42*).

### Thermodynamics

The molality of sulfuric acid, H_2_SO_4_ (mol kg^−1^), in an aqueous solvent is related to the weight fraction wt % H_2_SO_4_ by the equation

$$M_{H_{2}SO_{4}}=\frac{1}{\mu_{H_{2}SO_{4}}}\frac{w_{H_{2}SO_{4}}}{1-w_{H_{2}SO_{4}}}$$ (1)

where μ_H_2_SO_4__ = 0.098 kg mol^−1^ is the molecular weight of sulfuric acid.

Equation 1 is applied to a recent model of the sulfuric acid concentrations as a function of atmospheric height (*51*), with consideration of a range of concentrations on either side of the model values based on observational uncertainties (*43*). The uncertainties were estimated by comparing the “low-range,” 30 ppm, and “mid-range,” ∼300 ppm, concentrations of water vapor, a range spanning one order of magnitude. We apply this order of magnitude uncertainty for in-cloud H_2_O vapor to estimate the uncertainty in the wt % H_2_O in the clouds, following the method of Hallsworth *et al.* (*58*).

With increasing sulfuric acid concentration, rhomboclase eventually becomes unstable relative to AFS (*35*). To estimate the location of this phase boundary as a function of temperature and sulfuric acid concentration through the Venusian atmosphere, we determined the standard state heat capacity of this reaction ( $\Delta C_{\text{Pr}}^{o}$ ) and assumed that it remained constant as a function of temperature. This assumption is necessary because although the heat capacity of rhomboclase has been determined as a function of temperature, the heat capacity of AFS has only been estimated at a single temperature value (*35*). Nevertheless, these constraints provide appropriate estimates for the temperature dependence of the rhomboclase-AFS phase boundary. Because the reaction between rhomboclase and AFS depends only on water activity, this calculation provides an estimate of the location of the rhomboclase-AFS phase boundary through the Venusian atmosphere if water activity values can be determined from sulfuric acid concentrations and temperatures. To do this, we used the E-AIM model (*59*) to convert estimates of sulfuric acid concentrations to water activity at a given temperature through the atmosphere. The distribution of H_2_SO_4_-H_2_O as a function of atmospheric height was adapted from a condensation model detailed by Dai *et al.* (*51*). The calculated results show that rhomboclase is thermodynamically stable across several kilometers of the Venusian atmosphere and would be expected to eventually convert to AFS at sulfuric acid concentrations above approximately 75 wt % (Fig. 2). Our experimental observations show that the kinetics of such conversion is slow in acid concentrations below ∼80 wt % (Fig. 1).

### UV absorbance

A laser-driven light source (200 to 800 nm) EQ-99X-FC was aligned toward the fiber end of a Flame-S OceanInsight Spectrometer with a measuring wavelength window of 200 to 800 nm. Solution samples with suspended minerals were pipetted in a hermetically sealed Hellma 110-QS quartz cuvette, which provided a 1-mm path length for the diverging beam. The quartz cuvette was mounted in a Thorlabs cuvette holder together with the fiber end of the spectrometer on a controlled track, allowing distance adjustment between the sample and the light source to achieve maximum intensity without saturating the spectrometer.

Initial materials were prepared by suspending synthetic rhomboclase in a range of concentrations of sulfuric acid. Over a period of 30 days, rhomboclase in acid concentrations above ∼75 wt % has converted into AFS (Table 2 and Fig. 1). Optically optimum amounts of stable mineral phases suspended within their respective sulfuric acid concentrations were examined for their UV absorbance using OceanView software. Relative UV absorbance was presented by setting the maximum absorbance between 200 and 500 nm to unity, in the same manner as the MESSENGER/MASCS observations presented by Pérez-Hoyos *et al.* (*17*), and 2020 reflectance observations of Lee *et al.* (*18*) for ease of comparison. The original data showing optical depth and extinction coefficients as a function of wavelength can be found in the Supplementary Materials.

**Table 2.** Stability of rhomboclase. Resulting mineralogy after a 1-month suspension of rhomboclase (rbc) in varying concentrations of sulfuric acid. Acid concentration calculation takes into account H_2_O released from mineral transformation. Rhomboclase is stable up to concentrations of 74.2 wt % and transforms into acid ferric sulfate (AFS) within hours to days in higher concentrations (Fig. 1). It is observed that copiapite transforms into rhomboclase within 9 days in 51.3 wt % sulfuric acid (fig. S6) and follows the same rhomboclase to AFS transformation in higher concentrations.

*T* = 298.15, *P* = 1 bar				
Ex.No	H_2_SO_4_ wt %	Solution (g)	Solid (g)	Resulting mineralogy (XRD and Raman)
rbc-1	51.3	5.9916	0.5453	rbc
rbc-2	74.2	6.7096	0.4445	rbc
rbc-3	79.1	6.8232	0.3000	AFS
rbc-4	83.5	6.9012	0.2792	AFS
rbc-5	90.1	6.5734	0.0611	AFS
rbc-6	97.4	7.2205	0.2735	AFS

Once the extinction coefficients are determined for the liquid and solid phases, we use a root mean square goodness of fit with all combinations of liquids and solids in different relative fractions. This practice takes into account that the observed UV absorbance in the Venusian clouds may encompass the transition zone between rhomboclase and AFS, that is, between 55 and 65 km, or between 70 and 85 wt % H_2_SO_4_. We identified the best-fit absorbance to be a combination of 1 wt % rhomboclase in 74.2 wt % H_2_SO_4_ and 1.25 wt % AFS in 83.5 wt % H_2_SO_4_ (Fig. 3). See also the Supplementary Materials.

## Supplementary Materials

### This PDF file includes:

Supplementary Text Figs. S1 to S10 References
