# Supplementary material for: Iron-sulfur chemistry can explain the ultraviolet absorber in the clouds of Venus
Source: Sci Adv. 2024 Jan 3;10(1):eadg8826. doi: 10.1126/sciadv.adg8826 (PMC10776003; doi:10.1126/sciadv.adg8826)
Supplement: Supplementary file 1 — Supplementary Text Figs. S1 to S10 References [file sciadv.adg8826_sm.pdf]

Science Advances
AAAS

Supplementary Materials for

Iron-sulfur chemistry can explain the ultraviolet absorber in the clouds of Venus

Clancy Zhijian Jiang *et al.*

Corresponding author: Clancy Zhijian Jiang, zj259@cam.ac.uk; Paul B. Rimmer, pbr27@cam.ac.uk

Sci. Adv. **10**, eadg8826 (2024)
DOI: 10.1126/sciadv.adg8826

This PDF file includes:

Supplementary Text
Figs. S1 to S10
References

Supplementary Material

Absorbance Measurements

We prepared the samples as shown in Supplementary Figure 5. For each of the samples, we pipetted out 250 μL of solution (near the top of the liquid in the vial) and 2 μL of precipitate (near the bottom of the vial). The green arrows and text in Supplementary Figure 5 shows where the samples were taken. Solution and precipitate were then added to a Hellma 110-QS quartz cuvette with 1 mm pathlength and mixed. The optical depth for the solution with no added precipitate, τ_ℓ, is shown in Supplementary Figure 6, and the optical depth with the precipitate, $\tau_{s+\ell}$, is shown in Supplementary Figure 7.

We then calculate the extinction coefficient for the liquid (cm^2 g^{-1}), which is:

$$\varepsilon_\ell = \frac{\tau_\ell}{\rho L}, \tag{2}$$

where ρ (g cm^{-3}) is the density of the solution, based on the known wt% H_2SO_4, and $L = 0.1$ cm is the path length of the cuvette. The results for ε_ℓ are shown in Supplementary Figure 8.

To determine the extension coefficient for the solid, we take the difference in the optical depths:

$$\varepsilon_s = \frac{\tau_{s+\ell} - \tau_\ell}{\rho_s f_s L}, \tag{3}$$

where ρ_s (g cm^{-3}) is the mass density of the solid, either rhomboclase (2.23 g cm^{-3}), acid ferric sulfate (2.8 g cm^{-3}) or anhydrous ferric sulfate (3.1 g cm^{-3}), and $f_s = 2/250$ is the volume fraction of the solid. We fit the result to a smoothed function. The data and smoothed function are both shown in Supplementary Figure 9 for acid and anhydrous ferric sulfate, and in Supplementary Figure 10 for rhomboclase.

The mass fraction of the solution that is rhomboclase or anhydrous ferric sulfate is:

$$C_s = \frac{2}{250}\,\frac{\rho_s}{\rho(\mathrm{H_2SO_4})}, \tag{4}$$

where C_s (g/g) is the mass fraction of rhomboclase or anhydrous ferric sulfate, $\rho(\mathrm{H_2SO_4}) = 1.8\,\mathrm{g\,cm^{-3}}$, and so $C_s = 1\,\mathrm{wt\%}$ for rhomboclase, $1.25\,\mathrm{wt\%}$ for acid ferric sulfate and $1.37\,\mathrm{wt\%}$ for anhydrous ferric sulfate.

Formation of Ferric Sulfate from Fe(III) in Sulfuric Acid

In a 100 mL flask, we added 20 mL 95 wt% H_2SO_4 to 4 mL ultra-pure H_2O to achieve a clear solution of 24 mL 90 wt% H_2SO_4. We then added 797 mg anhydrous $FeCl_3$ to the solution. The mixture instantly released a cloud of yellow gas, assumed to be HCl, and changed to an opaque yellow-white color. Within 1 hour, the yellow-white material settled to the bottom of the flask, forming a precipiate below a yellowish solution of presumably chlorinated sulfuric acid. The precipitate was analyzed and its Raman spectrum identifies it as acid ferric sulfate. It is likely there is also anhydrous ferric sulfate, but this cannot be so readily determined using Raman spectra, and cannot be straight-forwardly extracted without altering the mineralogy.

Proposed UV Absorbers

Below is an abbreviated list of UV absorbers that have been proposed in the literature that are linked with the abundance of Fe and/or sulfur species in the Venus atmosphere.

$FeCl_3$: Ferric chloride ($FeCl_3$) in the clouds was first proposed to explain the unknown UV absorber by Kuiper (*44*). Later experiments and models support that $FeCl_3$ fits the UV observations (*45*), and can explain several other features of the aerosol and surface chemistry (*19*). However, the absorbance of $FeCl_3$ does not fit MESSENGER/MASCS observations of the unknown UV absorber (*17*).

Amorphous Sulfur: Toon et al. (*20*) proposed solid amorphous sulfur as the unknown UV absorber. The amount of amorphous sulfur required to explain the integrated UV absorbance in the middle and upper clouds is difficult to reconcile with the mass loading of the clouds (*24*), and the spectral shape of the absorption does not fit the observations (*17*).

S_2O: Disfulfur monoxide (S_2O) has been proposed as the UV absorber for Venus, both as as solid (*60, 61*) and gas (*25*), and fits the mid- and long-wavelength UV spectrum decently (*17*), but not the short wavelength light, at least, based on absorbance from solid S_2O. Various models predict < 1 ppb mixing ratios of S_2O at the cloud tops and above the clouds (*3, 5, 6*), and it is unclear whether these concentrations are sufficient to explain the observations.

$(NH_4)_2S_2O_5$: Condensed ammonium pyrosulfate ($(NH_4)_2S_2O_5$) was proposed by Titov (*22*). The presence of this species in any appreciably quantity is difficult to reconcile with the oxidizing atmosphere of Venus, although it could conceivably be a byproduct of biological nitrogen fixation in the clouds (*62*). Nevertheless, its absorbance does not fit the observations (*17*).

OSSO: cis- and trans- OSSO vapor have recently been proposed as candidates for the unknown UV absorber by Frandsen et al. (*23*). The UV absorbance of OSSO matches the unknown UV absorber very well (*17*), but requires amounts of OSSO that are difficult to reproduce with chemical kinetics models (*63*), and seem incompatible with observational constraints of OSSO concentrations (*15*).

Life: Life floating in the clouds is a potential explanation for the UV absorber (*64*). This hypothesis requires the presence of living cells that correlate with the heterogeneity of the UV absorber. The confirmation of the presence of life in the clouds of Venus will require several *in situ* missions, along with laboratory investigations into the potential for biological mechanisms in concentrated sulfuric acid with very low water activity. The upcoming Morning Star missions, such as the RocketLab probe (*65, 66*), is one such mission. Life as we know it would incorporate both iron and sulfur, and so this hypothetical absorber is also related to iron-sulfur

chemistry.

Supplementary Material Figures

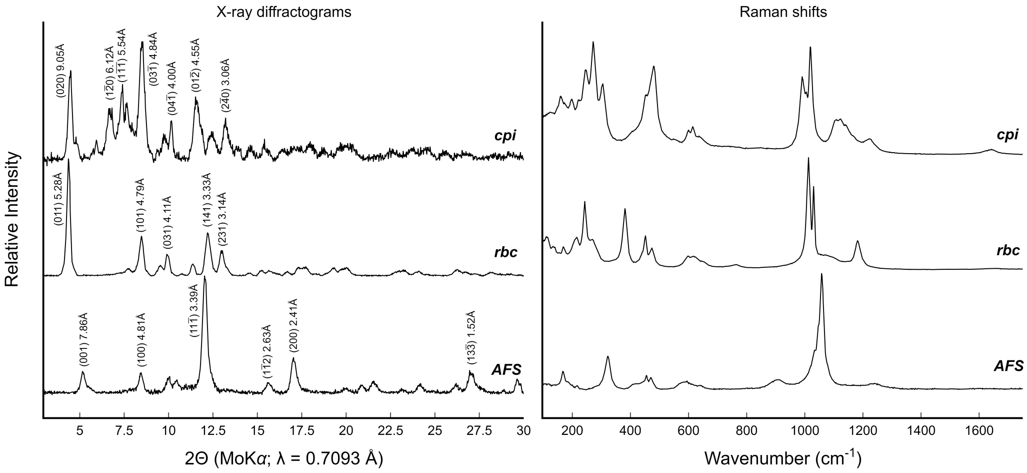

Figure S1: Powdered X-Ray Diffractograms (background removed) and Raman shift of copiapite (*cpi*), rhomboclase (*rbc*), and acid ferric sulfate (*AFS*). X-ray diffractograms were obtained with a Mo Kα (λ = 0.70930 Å), scanned between 2.5 to 30 deg 2Θ at a step size of 0.02 deg. Rietveld refinement results (Supplementary Fig. 4) confirm matching structures with reported copiapite (*32*), rhomboclase (*32*), and AFS (*42*). Raman shifts were obtained with a 532 nm laser at 50x magnification at 10 mW, and agree well with previous spectroscopic studies (*36, 38*).

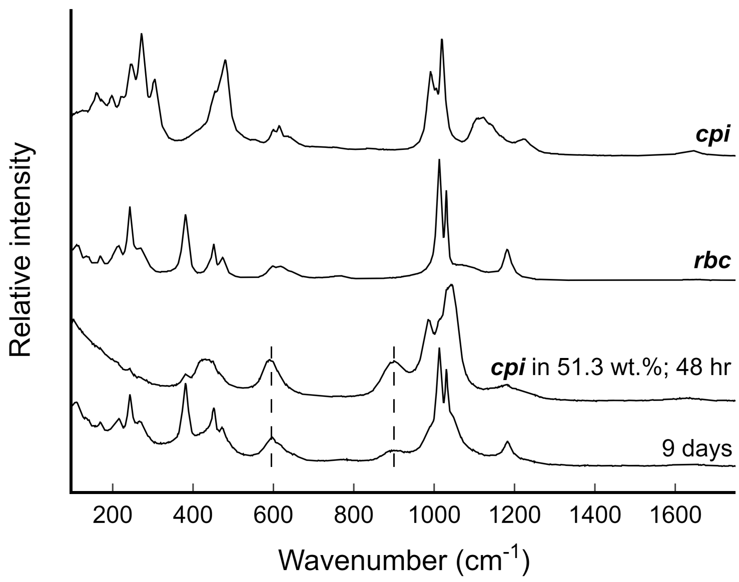

Figure S2: Raman shifts of copiapite (*cpi*), rhomboclase (*rbc*), and direct measurement of copiapite suspended in 51.3 wt% sulfuric acid after 48 and 216 hours. It is observed that copiapite has completely transformed into rhomboclase within 9 days, the rate of transformation is significantly faster in higher wt% of sulfuric acid. dashed lines indicate contribution by sulfuric acid

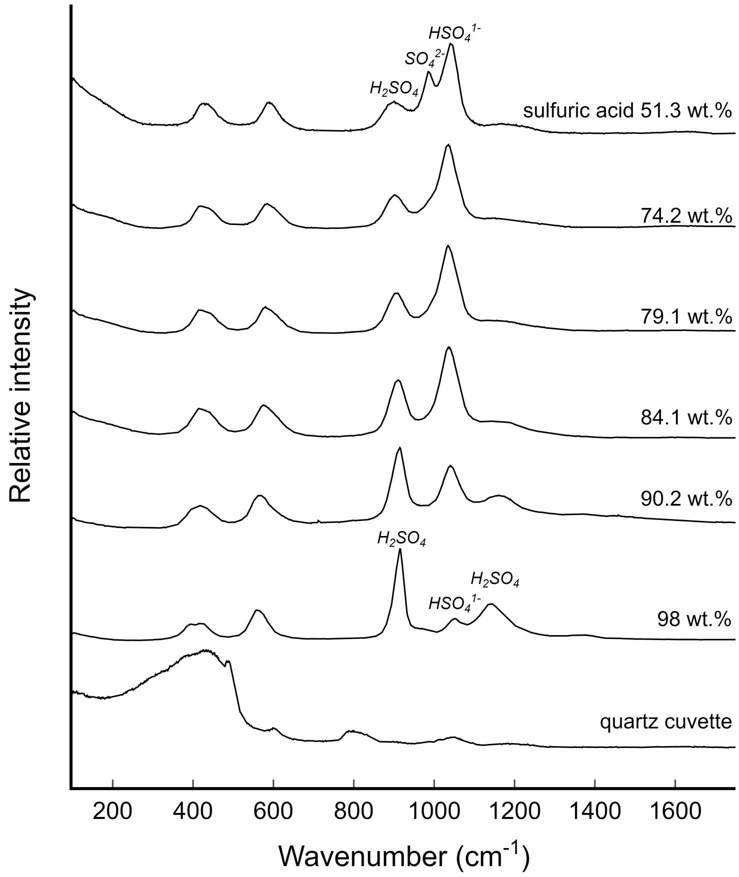

Figure S3: Raman shifts of sulfuric acid (H_2SO_4), bisulfate (HSO_4^-), and sulfate (SO_4^{2-}) for different wt% H_2SO_4. Also included are the Raman shifts for the quartz cuvette itself (fused silica). Of note are the changes in peak location and intensity with different wt% H_2SO_4, due in part to the change in ion concentrations, related to the $pKa_{1,2}$ of sulfuric acid.

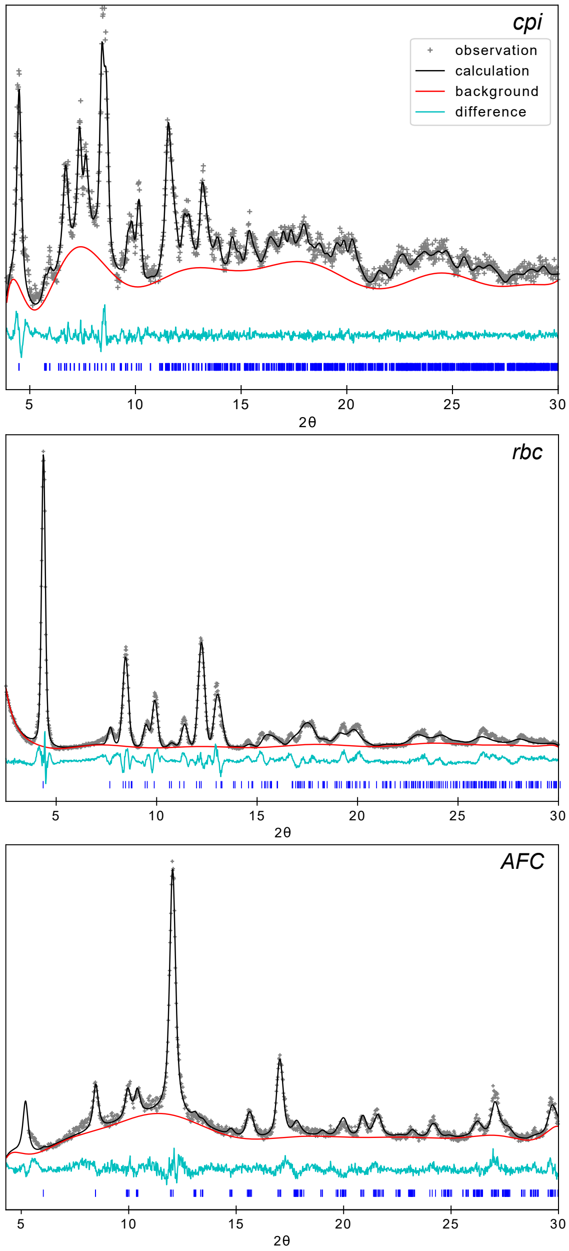

Figure S 4: Rietveld refinement of copiapite (*cpi*), rhomboclase (*rbc*) and acid ferric sulfate (*AFS*). Crystal structural information of copiapite (*32*), rhomboclase (*32*), and AFS (*42*) are downloaded from *American Mineralogist Crystal Structure Database* (http://rruff.geo.arizona.edu/AMS/amcsd.php), refinement achieved using GASA II software (*67*).

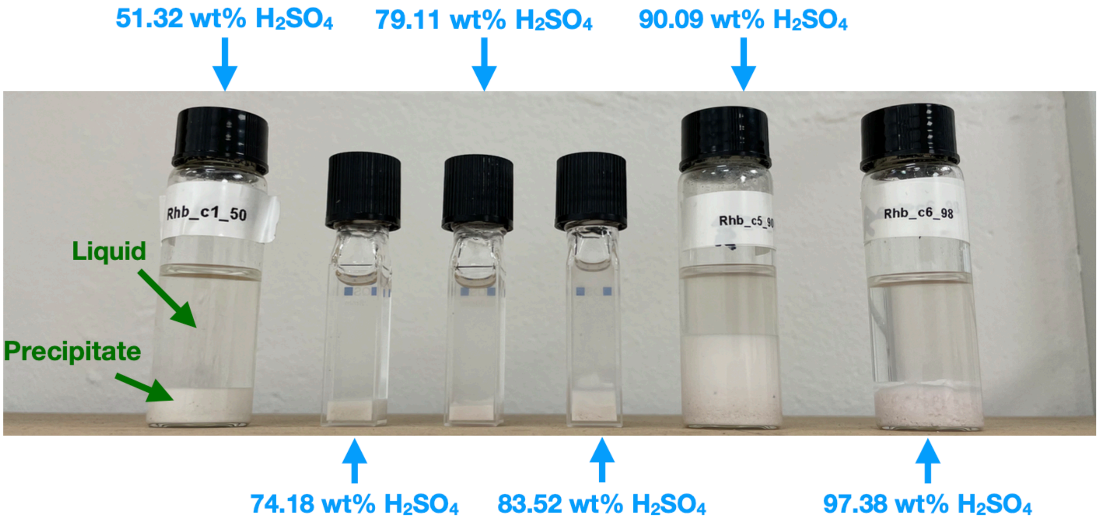

Figure S5: Mineral Samples. The various samples prepared, starting with rhomboclase added to various concentrations of sulfuric acid, as listed above (blue text and arrows). Precipitates form in all mixtures shown. The precipitate and solution are labelled for one of the samples with green text and arrows.

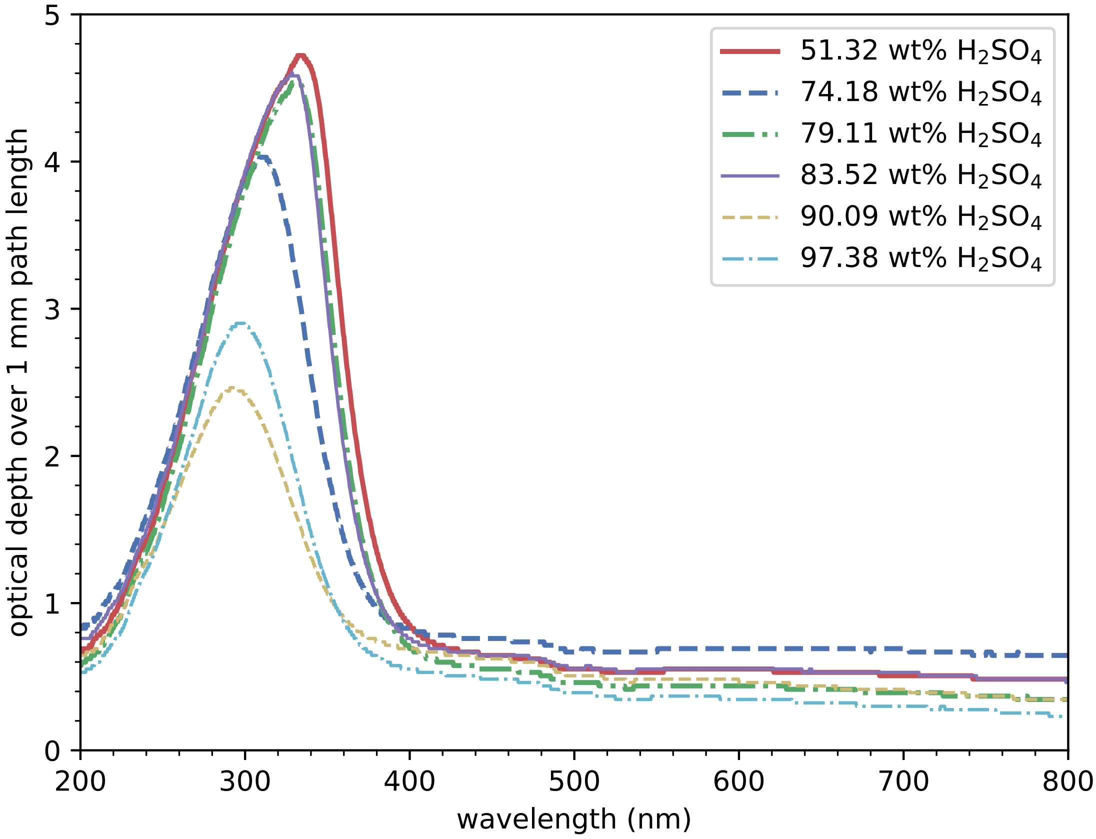

Figure S6: Liquid Optical Density Measurements. Optical density over a 1 mm path length as a function of wavelength for the liquid portion of the samples starting with rhomboclase, over a range of sulfuric acid concentrations.

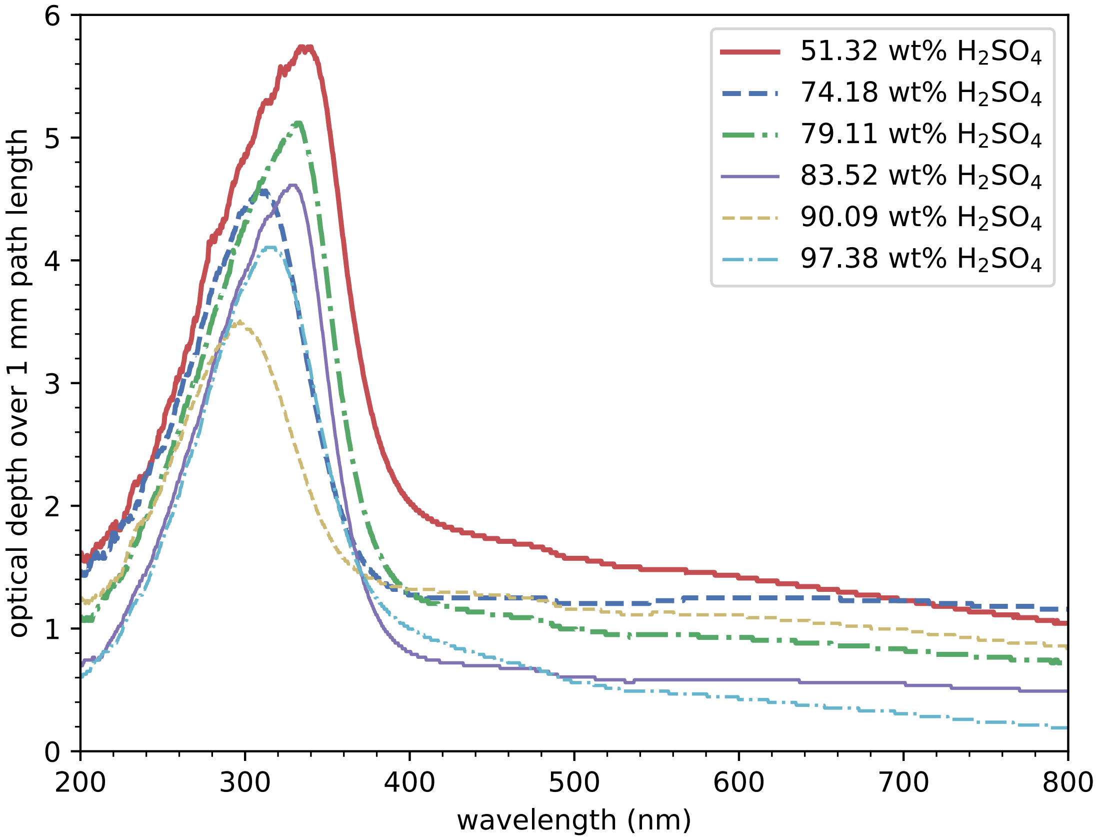

Figure S7: Optical Density of Solid Suspended in Liquid. Optical density over a 1 mm path length as a function of wavelength for the liquid portion (250 μL) with suspended solid (2 μL), starting with rhomboclase, over a range of sulfuric acid concentrations.

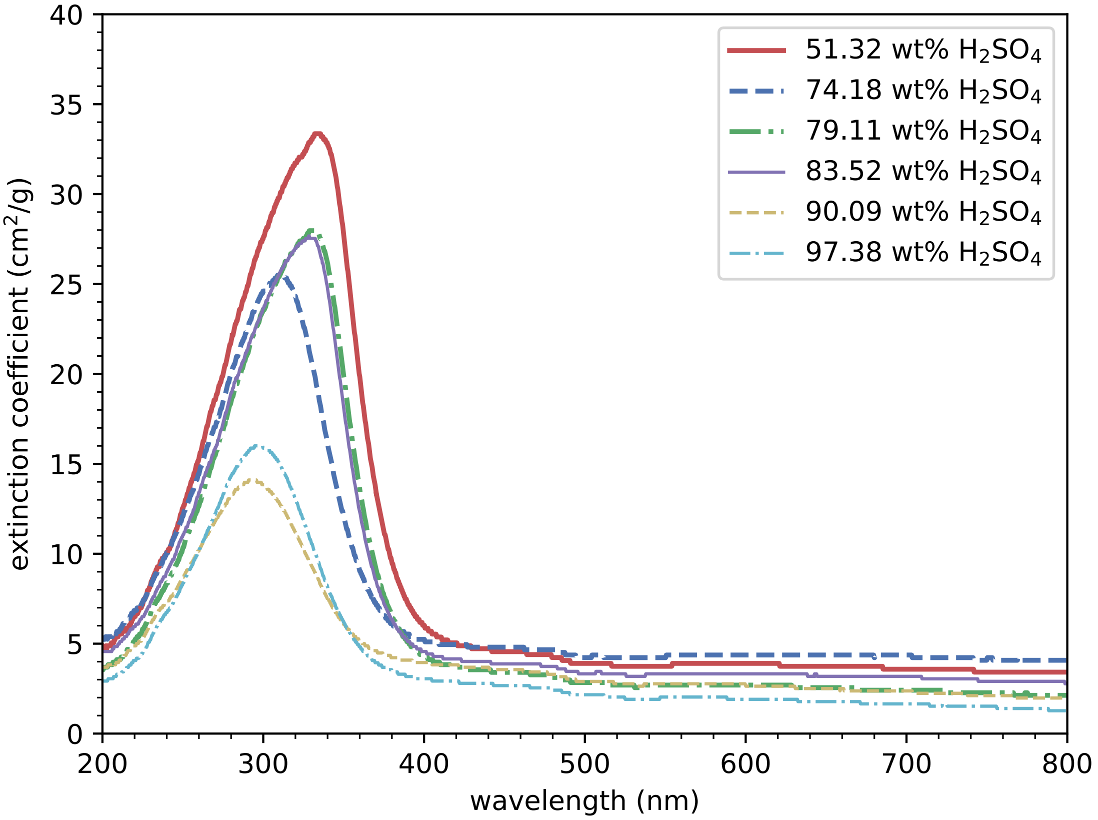

Figure S8: Liquid Extinction Coefficient. Extinction coefficient ($cm^2\ g^{-1}$) as a function of wavelength for the liquid portion of samples, starting with rhomboclase, over a range of sulfuric acid concentrations. Calculation is described in the SI.

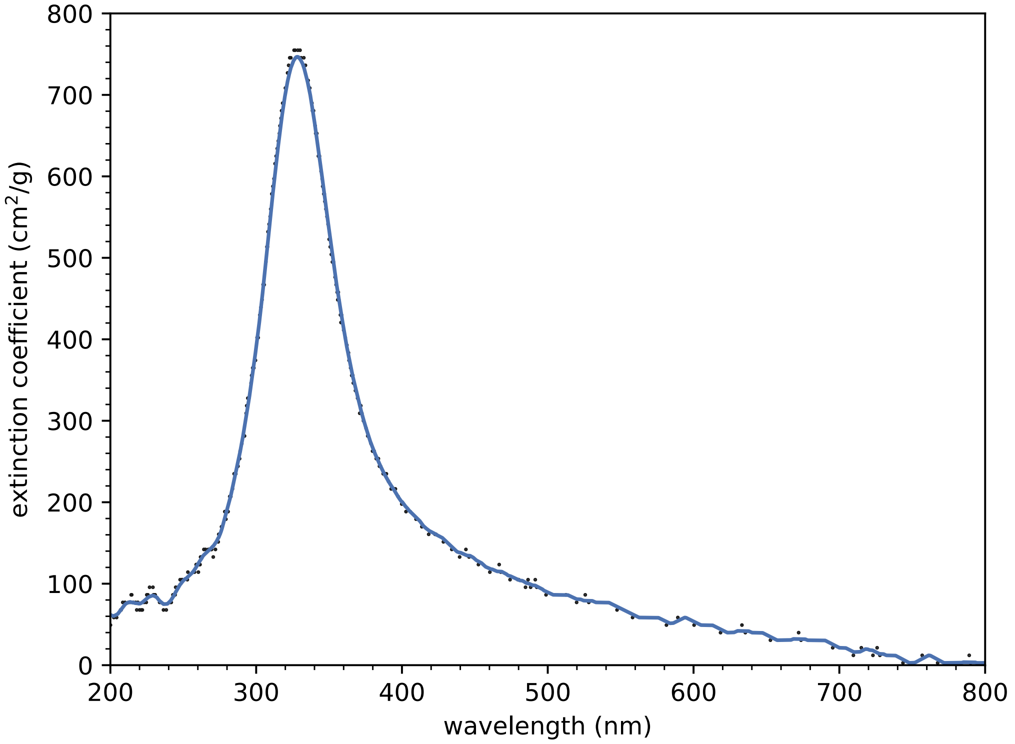

Figure S9: Extinction Coefficient for acid ferric sulfate. Extinction coefficient (cm^2 g^{-1}) as a function of wavelength for acid ferric sulfate ($(H_3O)Fe(SO_4)_2$) and possibly anhydrous ferric sulfate ($Fe_2(SO_4)_3$), calculated as described in the SI.

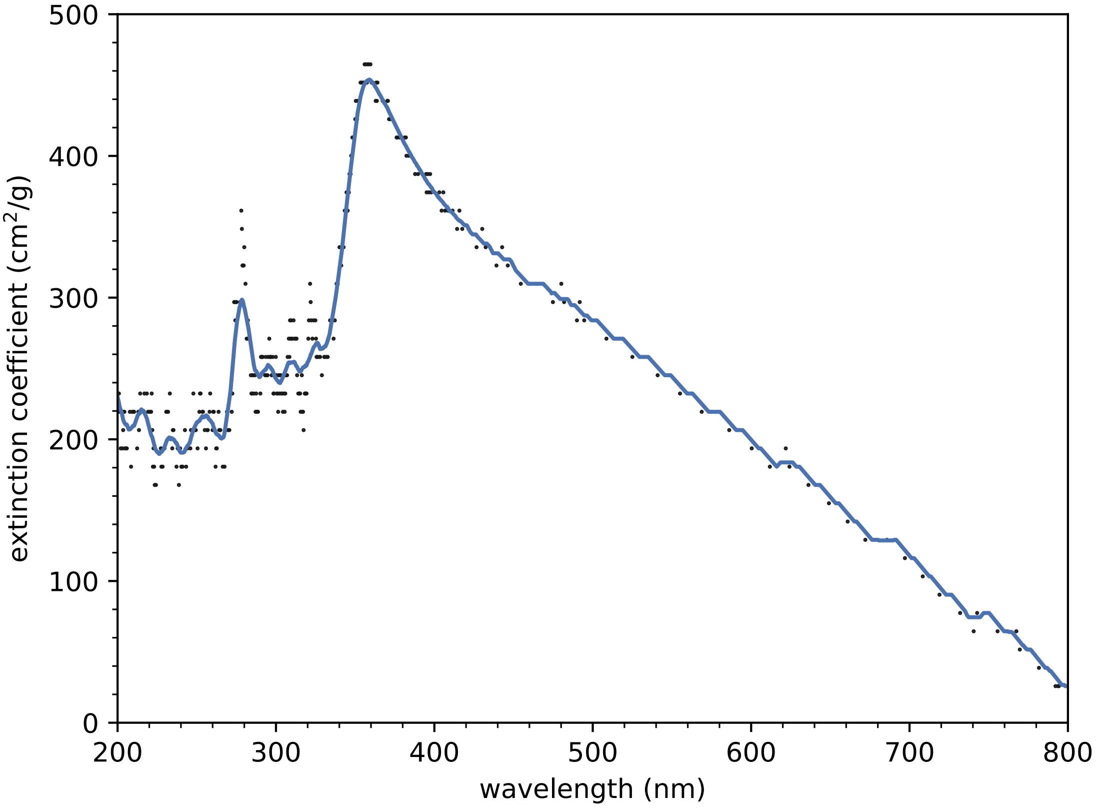

Figure S10: Extinction Coefficient for Rhomboclase. Extinction coefficient (cm^2 g^{-1}) as a function of wavelength for rhomboclase ($(H_5O_2)Fe(SO_4)_2 \cdot 3\,H_2O$), calculated as described in the SI.

REFERENCES AND NOTES

1. D. V. Titov, N. I. Ignatiev, K. McGouldrick, V. Wilquet, C. F. Wilson, Clouds and Hazes of Venus. *Space Sci. Rev.* **214**, 126 (2018).

2. V. A. Krasnopolsky, Chemical kinetic model for the lower atmosphere of Venus. *Icarus* **191**, 25–37 (2007).

3. X. Zhang, M. C. Liang, F. P. Mills, D. A. Belyaev, Y. L. Yung, Sulfur chemistry in the middle atmosphere of Venus. *Icarus* **217**, 714–739 (2012).

4. J. S. Greaves, A. M. S. Richards, W. Bains, P. B. Rimmer, H. Sagawa, D. L. Clements, S. Seager, J. J. Petkowski, C. Sousa-Silva, S. Ranjan, E. Drabek-Maunder, H. J. Fraser, A. Cartwright, I. Mueller-Wodarg, Z. Zhan, P. Friberg, I. Coulson, E. Lee, J. Hoge, Phosphine gas in the cloud decks of Venus. *Nat. Astron.* **5**, 655–664 (2021).

5. C. J. Bierson, X. Zhang, Chemical cycling in the Venusian atmosphere: A full photochemical model from the surface to 110 km. *J. Geophys. Res. Planets* **125**, e06159 (2020).

6. P. B. Rimmer, S. Jordan, T. Constantinou, P. Woitke, O. Shorttle, R. Hobbs, A. Paschodimas, Hydroxide salts in the clouds of Venus: Their effect on the sulfur cycle and cloud droplet pH. *Planet. Sci. J.* **2**, 133 (2021).

7. J. B. Garvin, S. A. Getty, G. N. Arney, N. M. Johnson, E. Kohler, K. O. Schwer, M. Sekerak, A. Bartels, R. S. Saylor, V. E. Elliott, C. S. Goodloe, M. B. Garrison, V. Cottini, N. Izenberg, R. Lorenz, C. A. Malespin, M. Ravine, C. R. Webster, D. H. Atkinson, S. Aslam, S. Atreya, B. J. Bos, W. B. Brinckerhoff, B. Campbell, D. Crisp, J. R. Filiberto, F. Forget, M. Gilmore, N. Gorius, D. Grinspoon, A. E. Hofmann, S. R. Kane, W. Kiefer, S. Lebonnois, P. R. Mahaffy, A. Pavlov, M. Trainer, K. J. Zahnle, M. Zolotov, Revealing the mysteries of Venus: The DAVINCI mission. *Planet. Sci. J.* **3**, 117 (2022).

8. Y. L. Yung, W. B. Demore, Photochemistry of the stratosphere of Venus: Implications for atmospheric evolution. *Icarus* **51**, 199–247 (1982).

9. F. E. Ross, Photographs of Venus. *Astrophys J.* **68**, 57 (1928).

10. R. G. Knollenberg, D. M. Hunten, The microphysics of the clouds of Venus—Results of the pioneer Venus particle size spectrometer experiment. *J. Geophys. Res.* **85**, 8039–8058 (1980).

11. V. A. Krasnopolsky, A sensitive search for nitric oxide in the lower atmospheres of Venus and Mars: Detection on Venus and upper limit for Mars. *Icarus* **182**, 80–91 (2006).

12. R. Mogul, S. S. Limaye, M. J. Way, J. A. Cordova, Venus' mass spectra show signs of disequilibria in the middle clouds. *Geophys. Res. Lett.* **48**, e91327 (2021).

13. B. Andreychikov, I. Akhmetshin, B. Korchuganov, L. Mukhin, B. Ogorodnikov, I. Petryanov, V. Skitovich, X-ray radiometric analysis of the cloud aerosol of Venus by the Vega 1 and 2 probes. *Cosmic Res.* **25**, 16 (1987).

14. T. Milojevic, A. H. Treiman, S. S. Limaye, Phosphorus in the clouds of Venus: Potential for bioavailability. *Astrobiology* **21**, 1250–1263 (2021).

15. E. Marcq, K. Lea Jessup, L. Baggio, T. Encrenaz, Y. J. Lee, F. Montmessin, D. Belyaev, O. Korablev, J.-L. Bertaux, Climatology of SO_2 and UV absorber at Venus' cloud top from SPICAV-UV nadir dataset. *Icarus* **335**, 113368 (2020).

16. Y. J. Lee, A. García-Muñoz, A. Yamazaki, M. Yamada, S. Watanabe, T. Encrenaz, Investigation of UV absorbers on Venus using the 283 and 365 nm phase curves obtained from Akatsuki. *Geophys. Res. Lett.* **48**, e90577 (2021).

17. S. Pérez-Hoyos, A. Sánchez-Lavega, A. García-Muñoz, P. G. J. Irwin, J. Peralta, G. Holsclaw, W. M. McClintock, J. F. Sanz-Requena, Venus upper clouds and the UV absorber from MESSENGER/MASCS observations. *J. Geophys. Res. Planets* **123**, 145–162 (2018).

18. Y. J. Lee, A. García Muñoz, A. Yamazaki, E. Quémerais, S. Mottola, S. Hellmich, T. Granzer, G. Bergond, M. Roth, E. Gallego-Cano, J.-Y. Chaufray, R. Robidel, G. Murakami, K. Masunaga, M. Kaplan, O. Erece, R. Hueso, P. Kabáth, M. Špoková, A. Sánchez-Lavega, M.-J. Kim, V. Mangano, K.-L. Jessup, T. Widemann, K.-I. Sugiyama, S. Watanabe, M. Yamada, T.

Satoh, M. Nakamura, M. Imai, J. Cabrera, Reflectivity of Venus's dayside disk during the 2020 observation campaign: Outcomes and future perspectives. *Planet. Sci. J.* **3**, 209 (2022).

19. V. A. Krasnopolsky, On the iron chloride aerosol in the clouds of Venus. *Icarus* **286**, 134–137 (2017).

20. O. B. Toon, R. P. Turco, J. B. Pollack, The ultraviolet absorber on Venus: Amorphous sulfur. *Icarus* **51**, 358–373 (1982).

21. W.-J. Lo, Y.-J. Wu, Y.-P. Lee, Ultraviolet absorption spectrum of cyclic S_2O in solid Ar. *J. Phys. Chem. A* **107**, 6944–6947 (2003).

22. D. V. Titov, On the possibility of aerosol formation in the chemical reaction between SO_2 and NH_3 under Venus atmosphere conditions. *Kosmicheskie Issledovaniia* **21**, 401–409 (1983).

23. B. N. Frandsen, P. O. Wennberg, H. G. Kjaergaard, Identification of OSSO as a near-UV absorber in the Venusian atmosphere. *Geophys. Res. Lett.* **43**, 11146–11155 (2016).

24. V. A. Krasnopolsky, Sulfur aerosol in the clouds of Venus. *Icarus* **274**, 33–36 (2016).

25. C. Y. Na, L. W. Esposito, Is disulfur monoxide a second absorber on Venus? *Icarus* **125**, 364–368 (1997).

26. I. Petrianov, B. Andreichikov, B. Korchuganov, E. Ovsiankin, B. Ogorodnikov, V. Skitovich, V. Khristianov, Iron in the clouds of venus. *Akademiia Nauk SSSR Doklady* **260**, 834–836 (1981).

27. M. Y. Zolotov, R. Mogul, S. S. Limaye, M. J. Way, J. B. Garvin, Venus cloud composition suggested from the pioneer Venus large probe neutral mass spectrometer data. *LPI Contributions* (2023), p. 2880.

28. L. M. Mukhin, D. F. Nenarokov, N. V. Porschnev, V. B. Bondarev, B. G. Gelman, G. Israèl, F. Raulin, J. Runavot, R. Thomas, Preliminary calibration results of VEGA 1 and 2 SIGMA-3 gas chromatograph. *Adv. Space Res.* **7**, 329–335 (1987).

29. G. A. Rowland, L. F. Phillips, Cloud photochemistry and its effect on the composition of the upper atmosphere of venus. *Geophys. Res. Lett.* **27**, 3301–3304 (2000).

30. G. Rowland, R. Van Eldik, L. Phillips, Photochemistry of concentrated sulfuric acid in the presence of SO_2 and FE (II), and implications for the cloud chemistry of venus. *J. Photochem. Photobiol. A Chem.* **153**, 1–10 (2002).

31. V. Moroz, Stellar magnitude and albedo data of venus, *in Venus, Hunten D. M., Ed. (University of Arizona Press, 1983), pp.* 27–35.

32. J. Majzlan, A. Navrotsky, R. B. McCleskey, C. N. Alpers, Thermodynamic properties and crystal structure refinement of ferricopiapite, coquimbite, rhomboclase, and $Fe_2(SO_4)_3(H_2O)_5$. *Eur. J. Mineral.* **18**, 175–186 (2006).

33. J. Majzlan, R. Michallik, The crystal structures, solid solutions and infrared spectra of copiapite-group minerals. *Mineral. Mag.* **71**, 553–569 (2007).

34. J. Majzlan, Advances and gaps in the knowledge of thermodynamics and crystallography of acid mine drainage sulfate minerals. *CHIMIA Int. J. Chem.* **64**, 699–704 (2010).

35. J. Majzlan, K.-D. Grevel, B. Kiefer, U. G. Nielsen, E. Grube, E. Dachs, A. Benisek, M. A. White, M. B. Johnson, Thermodynamics and crystal chemistry of rhomboclase, $(H_5O_2)Fe(SO_4)_2.2H_2O$, and the phase $(H_3O)Fe(SO_4)_2$ and implications for acid mine drainage. *Am. Mineral.* **102**, 643–654 (2017).

36. Z. C. Ling, A. Wang, A systematic spectroscopic study of eight hydrous ferric sulfates relevant to Mars. *Icarus* **209**, 422–433 (2010).

37. A. Wang, Z. C. Ling, Ferric sulfates on mars: A combined mission data analysis of salty soils at gusev crater and laboratory experimental investigations. *J. Geophys. Res.* **116**, E00F17 (2011).

38. A. Wang, Z. Ling, J. J. Freeman, W. Kong, Stability field and phase transition pathways of hydrous ferric sulfates in the temperature range 50°C to 5°C: Implication for martian ferric sulfates. *Icarus* **218**, 622–643 (2012).

39. N. J. Tosca, A. H. Knoll, S. M. McLennan, Water activity and the challenge for life on early Mars. *Science* **320**, 1204–1207 (2008).

40. C. de Bergh, V. I. Moroz, F. W. Taylor, D. Crisp, B. Bézard, L. V. Zasova, The composition of the atmosphere of Venus below 100km altitude: An overview. *Planet. Space Sci.* **54**, 1389–1397 (2006).

41. W. Xu, J. B. Parise, J. Hanson, $(H_3O)Fe(SO_4)_2$ formed by dehydrating rhomboclase and its potential existence on mars. *Am. Mineral.* **95**, 1408–1412 (2010).

42. R. C. Peterson, E. Valyashko, R. Wang, The atomic structure of $(H_3O)Fe^{3+}(SO_4)_2$ and rhomboclase, $(H_5O_2)Fe^{3+}(SO_4)_2 \cdot 2H_2O$. *Can. Mineral.* **47**, 625–634 (2009).

43. N. I. Ignatiev, V. I. Moroz, B. E. Moshkin, A. P. Ekonomov, V. I. Gnedykh, A. V. Grigoriev, I. V. Khatuntsev, Water vapour in the lower atmosphere of venus: A new analysis of optical spectra measured by entry probes. *Adv. Space Res.* **19**, 1159–1168 (1997).

44. G. P. Kuiper, On the nature of the Venus clouds, in *Symposium-International Astronomical Union*, C. Sagan, T. Owen, H. J. Smith, Eds. (Cambridge Univ. Press, 1971), pp. 91–109.

45. L. V. Zasova, V. A. Krasnopolskii, V. I. Moroz, Vertical distribution of SO_2 in upper cloud layer of Venus and Origin of U.V.-absorption. *Adv. Space Res.* **1**, 13–16 (1981).

46. S. Seager, J. J. Petkowski, P. Gao, W. Bains, N. C. Bryan, S. Ranjan, J. Greaves, The Venusian lower atmosphere haze as a depot for desiccated microbial life: A proposed life cycle for persistence of the Venusian aerial biosphere. *Astrobiology* **21**, 1206–1223 (2021).

47. V. A. Krasnopolsky, Vertical profiles of H_2O, H_2SO_4, and sulfuric acid concentration at 45–75km on Venus. *Icarus* **252**, 327–333 (2015).

48. J. K. Barstow, C. C. C. Tsang, C. F. Wilson, P. G. J. Irwin, F. W. Taylor, K. McGouldrick, P. Drossart, G. Piccioni, S. Tellmann, Models of the global cloud structure on Venus derived from Venus Express observations. *Icarus* **217**, 542–560 (2012).

49. G. Arney, V. Meadows, D. Crisp, S. J. Schmidt, J. Bailey, T. Robinson, Spatially resolved measurements of H_2O, HCl, CO, OCS, SO_2, cloud opacity, and acid concentration in the Venus near-infrared spectral windows. *J. Geophys. Res. Planets* **119**, 1860–1891 (2014).

50. W. J. Markiewicz, E. V. Petrova, O. S. Shalygina, Aerosol properties in the upper clouds of Venus from glory observations by the Venus Monitoring Camera (Venus Express mission). *Icarus* **299**, 272–293 (2018).

51. L. Dai, X. Zhang, W. D. Shao, C. J. Bierson, J. Cui, A simple condensation model for the H_2SO_4-H_2O gas-cloud system on venus. *J. Geophys. Res. Planets* **127**, e2021JE007060 (2022).

52. M. C. Sauer, I. A. Shkrob, R. Lian, R. A. Crowell, D. M. Bartels, X. Chen, D. Suffern, S. E. Bradforth, Electron photodetachment from aqueous anions. 2. Ionic strength effect on geminate recombination dynamics and quantum yield for hydrated electron. *J. Phys. Chem. A* **108**, 10414–10425 (2004).

53. M. Y. Zolotov, Iron salts and oxides in the history of the surface-atmosphere-cloud system on Venus, in *52nd Lunar and Planetary Science Conference* (2021), p. 2615.

54. J. D. Carrillo-Sánchez, D. Nesvorný, P. Pokorný, D. Janches, J. M. C. Plane, Sources of cosmic dust in the Earth’s atmosphere. *Geophys. Res. Lett.* **43**, 11979–11986 (2016).

55. K. H. Baines, D. Nikolić, J. A. Cutts, M. L. Delitsky, J.-B. Renard, S. M. Madzunkov, L. M. Barge, O. Mousis, C. Wilson, S. S. Limaye, N. Verdier, Investigation of Venus cloud aerosol and gas composition including potential biogenic materials via an aerosol-sampling instrument package. *Astrobiology* **21**, 1316–1323 (2021).

56. C. M. Pieters, J. W. Head, W. Patterson, S. Pratt, J. Garvin, V. L. Barsukov, A. T. Basilevsky, I. L. Khodakovsky, A. S. Selivanov, A. S. Panfilov, Y. M. Gektin, Y. M. Narayeva, The color of the surface of Venus. *Science* **234**, 1379–1383 (1986).

57. S. M. Kane, R. S. Timonen, M. T. Leu, Heterogeneous chemistry of acetone in sulfuric acid solutions: Implications for the upper troposphere. *J. Phys. Chem. A* **103**, 9259–9265 (1999).

58. J. E. Hallsworth, T. Koop, T. D. Dallas, M.-P. Zorzano, J. Burkhardt, O. V. Golyshina, J. Martín-Torres, M. K. Dymond, P. Ball, C. P. McKay, Water activity in Venus's uninhabitable clouds and other planetary atmospheres. *Nat. Astron.* **5**, 665–675 (2021).

59. A. S. Wexler, S. L. Clegg, Atmospheric aerosol models for systems including the ions H^+, NH_4^+, Na^+, SO_4^{2-}, NO_3^-, Cl^-, Br^-, and H_2O. *J. Geophys. Res. Atmos.* **107**, 4207 (2002).

60. B. H. Toby, R. B. V. Dreele, *GSAS-II*: The genesis of a modern open-source all purpose crystallography software package. *J. Appl. Cryst.* **46**, 544–549 (2013).
